# Optimization of Micro-Pollutants’ Removal from Wastewater Using Agricultural Waste-Derived Sustainable Adsorbent

**DOI:** 10.3390/ijerph182111506

**Published:** 2021-11-01

**Authors:** Areej Alhothali, Tahir Haneef, Muhammad Raza Ul Mustafa, Kawthar Mostafa Moria, Umer Rashid, Kashif Rasool, Omaimah Omar Bamasag

**Affiliations:** 1 Department of Computer Sciences, Faculty of Computing and Information Technology, King Abdulaziz University, Jeddah 21589, Saudi Arabia; aalhothali@kau.edu.sa (A.A.); kmoria@kau.edu.sa (K.M.M.); obamasek@kau.edu.sa (O.O.B.); 2 Department of Civil and Environmental Engineering, Universiti Teknologi PETRONAS, Seri Iskandar 32610, Perak, Malaysia; 3 Centre for Urban Resource Sustainability, Institute of Self-Sustainable Building, Universiti Teknologi PETRONAS, Seri Iskandar 32610, Perak, Malaysia; 4 Institute of Nanoscience and Nanotechnology (ION2), Universiti Putra Malaysia, Serdang 43400, Selangor, Malaysia; umer.rashid@upm.edu.my; 5 Qatar Environment and Energy Research Institute (QEERI), Hamad Bin Khalifa University (HBKU), Doha 5825, Qatar; krasool@hbku.edu.qa; 6 Center of Excellence in Smart Environment Research, King Abdulaziz University, Jeddah 21589, Saudi Arabia

**Keywords:** wastewater, micro-pollutants, palm kernel shell biochar, response surface methodology

## Abstract

Water pollution due to the discharge of untreated industrial effluents is a serious environmental and public health issue. The presence of organic pollutants such as polycyclic aromatic hydrocarbons (PAHs) causes worldwide concern because of their mutagenic and carcinogenic effects on aquatic life, human beings, and the environment. PAHs are pervasive atmospheric compounds that cause nervous system damage, mental retardation, cancer, and renal kidney diseases. This research presents the first usage of palm kernel shell biochar (PKSB) (obtained from agricultural waste) for PAH removal from industrial wastewater (oil and gas wastewater/produced water). A batch scale study was conducted for the remediation of PAHs and chemical oxygen demand (COD) from produced water. The influence of operating parameters such as biochar dosage, pH, and contact time was optimized and validated using a response surface methodology (RSM). Under optimized conditions, i.e., biochar dosage 2.99 g L^−1^, pH 4.0, and contact time 208.89 min, 93.16% of PAHs and 97.84% of COD were predicted. However, under optimized conditions of independent variables, 95.34% of PAH and 98.21% of COD removal was obtained in the laboratory. The experimental data were fitted to the empirical second-order model of a suitable degree for the maximum removal of PAHs and COD by the biochar. ANOVA analysis showed a high coefficient of determination value (R^2^ = 0.97) and a reasonable second-order regression prediction. Additionally, the study also showed a comparative analysis of PKSB with previously used agricultural waste biochar for PAH and COD removal. The PKSB showed significantly higher removal efficiency than other types of biochar. The study also provides analysis on the reusability of PKSB for up to four cycles using two different methods. The methods reflected a significantly good performance for PAH and COD removal for up to two cycles. Hence, the study demonstrated a successful application of PKSB as a potential sustainable adsorbent for the removal of micro-pollutants from produced water.

## 1. Introduction

The development of industrialization has led to a huge scale of water pollution across the globe. Water pollution poses a serious threat to aquatic life and human beings [1,2]. It contains several types of menacing contaminants such as dyes, bacteria, polycyclic aromatic hydrocarbons (PAHs), volatile organic compounds, viruses, and heavy metals. Amongst all other water pollutants, PAHs are hazardous organic compounds that are naturally non-biodegradable due to their chemical properties. PAHs can quickly accumulate in the human body and cause carcinogenic diseases such as skin cancer, kidney failure, ulcers, brain damage, hepatitis, and liver damage [3]. These refractory organic pollutants are also present in oil and gas extraction industries’ wastewater in considerable amounts [4]. The wastewater obtained from oil and gas exploration industries is known as produced water (PW) [5]. The range of PAH concentration in PW reported by various research is from 124 to 1000 μg L^−1^ [6,7,8,9], and these pollutants are precarious even in low concentrations (μg L^−1^–mg L^−1^) [7]. In addition, the United States Environmental Protection Agency has declared 16 PAHs as priority chemicals because of their mutagenic and carcinogenic effects [3]. Globally, approximately half a million tons of PW are produced from oil and gas exploration industries [10]. Discharging PW without proper treatment into water bodies can put millions of lives at severe risk of carcinogenic disease. Therefore, the removal of PAHs is necessary to protect the environment and human beings from the hazardous effect of PAHs.

Several treatment methods have been applied for removal of PAHs from wastewater such as photo-degradation, electrocoagulation, Fenton oxidation, heterogeneous Fenton oxidation, etc. (Table 1). However, these technologies have drawbacks, such as high operational costs, the production of toxic by-products, high energy consumption, and intricate designs. These limitations make the application of these methods disadvantageous and impractical. There is a need for new techniques and materials characterized by easier management, better performance, environmental friendliness, and cost effectiveness in terms of the removal of PAHs from wastewater.

In recent years, biochar, as an adsorbent derived from agriculture waste, has gained attention because of its environmentally friendly nature, low cost, ready availability, sizeable porous structure, simple use, and abundant functional groups [22,23]. Numerous researchers have employed biochar obtained from different agricultural wastes such as wheat straw, bamboo biomass, orange peels, paper mill sludge biochar, enteromorpha prolifera, corn stalk, soybean straw, and rice straw for the removal of PAHs from different kinds of wastewater and soils [24,25,26,27,28,29]. However, until now, no one has investigated the potential of palm kernel shell biochar (PKSB) for the removal of PAHs. Thus far, few scientific research studies are reported in the literature on the usage of PKSB for the adsorption of fluoride [30], heavy metals [31,32], crystal violet [33], and phenol [34] regarding efficient removal performance [35]. The positive outcomes of PKSB for different applications encourage investigating its potential for the removal of PAHs from PW. Additionally, in Malaysia, palm kernel shell (PKS) is abundantly available, making it more economical than other agricultural waste available for biochar. PKS is more beneficial due to its novel properties such as low ash, high energy, and low sulfur contents. From 1.0 tons of palm oil, roughly 0.3 tons of PKS is produced during the milling of oil palm fruits. In Malaysia, PKS production is approximately 2.4 million tons per year [36]. Only 1–2% of PKS is used in energy and other commercial sectors, while reaming could be a potential source of biochar and needs to be estimated.

In this study, the application of PKSB as a potential absorbent for the removal of PAHs and chemical oxygen demand (COD) from wastewater was investigated. Additionally, response surface methodology (RSM) was used for the optimization process. This study provides a successful application of PKSB as a potential absorbent for the removal of COD and PAHs from wastewater, showing it to be a sustainable absorbent and efficient alternative to other available methods.

## 2. Materials and Methods

### 2.1. Reagents and Materials

PW samples were obtained from an oil and gas exploration site in the South East Asia region. Hydrochloric acid (HCl, 37%) and potassium hydroxide (KOH, 85%) were obtained from Sigma-Aldrich (M) Sdn. Bhd., Petaling Jaya Malaysia. Acetonitrile, dichloromethane, and sodium sulphate were purchased from R and M chemicals, IPOH Malaysia. Standard reference material, PAHs mix (2000 μg/mL) in dichloromethane was bought from Sigma-Aldrich (M) Sdn. Bhd., Petaling Jaya Malaysia. All the chemicals used in this study were analytical grade without any further purification.

### 2.2. PKSB Adsorbent Preparation

The PKS was obtained from Sarawak Oil Palms Berhad, Malaysia. The PKS was converted into powder using a grinder. The palm kernel shell acidic adsorbent was synthesized utilizing a sulfonating process (with some modifications from the previous procedure) after the pyrolysis of soaked PKS [23]. The calcination of soaked or unsoaked PKS was carried out with tube furnace at 400 °C in N_2_ atmosphere for 120 min until a black substance formed. The produced black material was washed extensively using hot distilled water at more than 80 °C until pH of black solid material (PKSB) reached up to pH 7. Then, it was placed into oven for one day at 80 °C for drying. After drying, the PKSB was crushed into powder form using mortar and pestle. In various previous studies, the PKSB was prepared and characterized before and after the treatment processes based on the study objectives [37,38,39]. The detailed synthesis process and the characterization of the PKSB can be studied from previous work [40,41]. The particle size range of the PKSB used in this study was less than the 250 μm, identified by Rashid et al. [40].

### 2.3. Analytical Methods

A glass conical flask (250 mL) was used as a reactor for laboratory-scale experiments. Concentrated HCl and/or NaOH were utilized for adjusting the pH of PW to the desired value before adding the adsorbent. The values of three factors, pH, COD, and PAHs, were measured immediately before and after each experiment following American Public Health Association (APHA) standard methods [42]. Then, 100 mL of PW was used in each run. The reactor was placed on a fixed rotary shaker (250 rpm) for agitation of the water sample and adsorbent for the desired reaction time. The pH of the aqueous sample was determined by a benchtop digital pH meter ST3100-B (OHAUS Corporation, Parsippany-Troy Hills, NJ, USA) calibrated with pH 4.0, 7.0, and 10.0 standard buffers. The COD of the water sample was measured by the colorimetric method via a spectrophotometer DR2800 (HACH Company, Loveland, CO, USA). The concentration of PAHs was measured via gas chromatography–mass spectrometry (Agilent, model G7035A, combination of 7820A GC system with 5977E (MSD, Santa Clara, CA, USA)) following a previously described procedure in our study [7]. All the tests were performed in triplicate, and average values were utilized to minimize errors. The percentage removal efficiency of PAHs or COD was achieved using Equation (1):

(1) $$\mathrm{Removal}\;(\%)=(Ci-\frac{Cf}{Ci})\;\times \;100$$

where *Ci* shows the initial concentration of PAHs or COD; *Cf* indicates the final concentration of PAHs or COD.

### 2.4. Experimental Design via Response Surface Methodology

For optimization purposes, a response surface methodology (RSM), based on a central composite design (CCD), was utilized with Design Expert software v11 (Stat-Ease, Minneapolis, MN, USA). Typically, RSM is used in a variety of statistical and mathematical approaches to optimize the effect of independent variables on responses. CCD is a standard and reliable RSM design to develop an experimental setup for simultaneous analysis and testing. CCD usually optimizes suitable factors with a minimum number of experiments and also evaluates the interaction between independent variables [43]. The complete layout matrix and responses to various independent variables were carried out using CCD. In this study, three independent variables were examined, namely biochar dosage (0.25–3.00 g/L), pH (4.0–10.0), and contact time (30–270 min), while the removal of PAHs and COD was considered as a dependent variable (responses). The overall experimental runs based on three independent parameters developed by CCD were 17 (=2^k^ + 2 k + 3), where k stands for the number of parameters (=3). In total, 17 experiments were carried out with 3 duplications at the center point to evaluate the pure error. All the responses obtained from the lab’s experiments were utilized to develop an empirical model (2nd order polynomial equation) associated with the interaction of parameters and the prediction of responses, as shown in Equation (2):

(2) $$y={\;\beta }_{0}+\sum^{\;}\beta_{i}x_{i}+\sum^{\;}\beta_{\mathrm{ii}}x_{i}^{2}+\sum^{\;}\beta_{\mathrm{ij}}x_{i\;}x_{j}$$

In Equation (2), y, β_0_, β_i_, β_ii_, and β_ij_ represent responses, constant coefficient, linear coefficient, quadratic coefficient, and interaction coefficient, respectively. Similarly, *x_i_* and *x_j_* are coded values of independent variables [44].

Furthermore, the statistical analysis and model fitting were analyzed using analysis of variance (ANOVA). ANOVA illustrates the significance of independent factors and their mutual interaction effect in the adsorption process. It also depicts the interaction between actual results and equations, which includes significance parameters and responses. In addition, the significance of the model is assessed by *F*-value and *p*-value. The higher *F*-value (>4.00) and lower *p*-value (<0.05) imply that the polynomial regression equation can address an almost complete variation in responses. A *p*-value less than 0.05 shows that the model is significant, while a *p*-value higher than 0.1 indicates the model is insignificant.

### 2.5. Regeneration Study

Regeneration study of PKSB was carried out by two different methods, namely microwave irradiation and water treatment, to evaluate and compare the potential of spent adsorbent for PAH and COD removal. The microwave treatment method used by Caliskan et al. [45] was practiced in the current study. Briefly, dielectric parameter testers/a microwave (AD-8320) manufactured by Püschner Microwave Power Systems, Germany were used for these purposes. The device is made up of three parts: a power source, a directional coupler, and a microwave receiver. Microwave heat is generated at 2450 MHz, with power varying from 0 to 3 kW. For measuring the temperature, a K-type thermocouple with shields was used. Mullite crucibles, with inner diameters of 90 and 120 mm, and with good heat-shock and wave-transparency properties, were used [46,47]. For microwave regeneration, biochar (10 g) was precisely weighed and inserted into a microwave reactor box. The effects of roasting temperatures (150 °C, 350 °C) and holding times (40, 60 min) were adjusted, while for water treatment, hot water with two different temperatures of 40 °C and 80 °C was used to wash the spent PKSB. Approximately 2.0 g of spent PKSB and 76 mL of deionized water were mixed in a glass beaker. After that, it was shaken for 5.0 h at 30 °C in a water bath shaker. After every 1 h interval, the deionized water was replaced and filtered. Then, the washed PKSB was placed in an oven and dried at 105 °C for 24 h. The same procedure was followed for PKSB regeneration with water at 80 °C.

The potential of regenerated PKSB was evaluated by subjecting it to the removal of PAHs and COD at optimized operating parameters. The removal–regeneration cycles were repeated for four cycles, and the removal of PAHs and COD of each cycle was measured. In addition, all the experiments were performed in triplicate to ensure accuracy and minimize the error in obtained results.

## 3. Results and Discussions

### 3.1. Produced Water Characterization and GC-MS Analysis for PAHs Concentration

**Figure 1:**
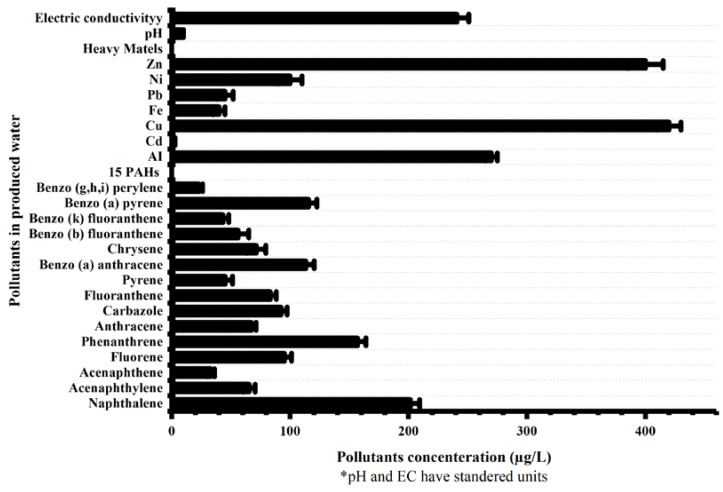
Characterization of produced water.

The PW was analyzed by pH [48], electrical conductivity (EC) [49], chloride (Cl^−^) [50], heavy metals [51], COD [52], and PAHs via standard methods [53]. The concentrations of different pollutants, such as heavy metals and PAHs, in PW are shown in Figure 1. The concentration of CI− in PW was 1400 mg/L, while GC-MS analysis quantified 15 PAHs in produced water, as shown in Figure 1. Total concentration of 15 PAHs (ΣPAHs) observed in PW was 1310 µg/L. As shown in Figure 1, among all 15 PAHs, the concentration of naphthalene (almost 200 µg/L) was higher than all other PAH components. The concentration of benzo (a) pyrene and benzo (a) anthracene was same, with 115 µg/L concentration in PW. Similarly, anthrathen and acenaphtylene were both 65 µg/L. However, benzo (g,h,i) perylene was observed in the lowest concentration (23 µg/L) compared to the other 15 PAHs. The high concentration of PAHs in PW needs to be removed prior to discharging PW into any nearby body of water.

### 3.2. Central Composite Design and Statistical Analysis

All 17 experimental runs of the CCD design matrix and the responses for PAH and COD removal are portrayed in Table 2. The values of responses were different for each experimental run based on their different values of independent variables. The results show (Table 2) that the maximum removal of PAHs (83.90%) was achieved at 1.62 g/L of biochar dosage, 4.0 pH, and 150 min of contact time, while the minimum removal of PAHs (40.20%) was obtained at 3.0 g/L of biochar dosage, 10.0 pH, and 30 min of contact time. Similarly, the maximum removal of COD (97.80%) was observed at 0.25 g/L biochar concentration, 10.0 pH, and 270 min of contact time, while the minimum COD removal (52.20%) was attained at 1.62 g/L adsorbent dosage, 7.0 pH, and 30 min reaction time. The experimental findings for PAH removal (y_1_) and COD removal (y_2_) were fitted to the second-order regression models, represented in Equations (3) and (4), respectively, in terms of coded independent variables:

(3) $$\begin{array}{c} y_{1}=68.38+2.85x_{1}-5.24x_{2}x_{i}^{2}+9.03x_{3}-5.66x_{1}x_{2}+0.48x_{1}x_{3}+8.21x_{2}x_{3}+\\ 7.18x_{1}^{2}+7.33x_{2}^{2}-16.02x_{3}^{2} \end{array}$$

(4) $$\begin{array}{c} y_{2}=69.51-2.45x_{1}-2.57x_{2}x_{i}^{2}+8.38x_{3}-4.24x_{1}x_{2}+4.01x_{1}x_{3}+\\ 6.66x_{2}x_{3}+1.06x_{1}^{2}+22.46x_{2}^{2}-7.79x_{3}^{2} \end{array}$$

In Equations (3) and (4) *x*_1_, *x*_2_, and *x*_3_ stand for biochar dosage, pH, and contact time, respectively. Negative signs (−) and positive signs (+) in theses equations indicate a variable’s synergistic and antagonistic effects [43].

The experimental results were then analyzed to evaluate the significance and suitability of the model using ANOVA. The model *F*-value of PAHs (25.77) and COD (38.06) removal are greater than the critical *F*-value of both models (0.33 and 0.99, respectively). Thus, these results demonstrate that both models are significant. Furthermore, the significance of PAH and COD models can also be assessed by *p*-value. The model terms of both models, such as *x*_1_, *x*_2_, *x*_3_, *x*_1_ *x*_2_, *x*_2_*x*_3,_ *x*_2_^2^, and *x*_3_^2^, are significant, as shown in Table 3. However, the *p*-values of the “Lack of Fit” for PAHs and COD models were 0.85 and 0.57, respectively, showing non-significance. Non-significant “Lack of Fit” is acceptable for the model [7,54], which implies that the quadratic regression model is significant/adequate to describe the relation between all three independent variables and responses (PAHs and COD).

**Figure 2:**
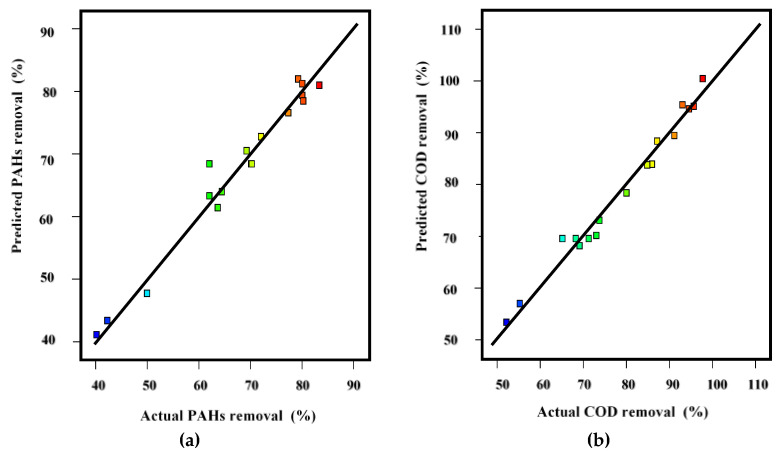
Predicated and experimental removal: (**a**) PAH removal and (**b**) COD removal.

Furthermore, the significance of the PAHs and COD models was also verified by the coefficient of variation (C.V.), adequate precision (AP), coefficient of determination (R^2^), and standard deviation (SD), as shown in Table 4. In this case, the R^2^ values of PAHs and COD models were 0.97 and 0.96, respectively. Furthermore, the R^2^ values of both models were close to unity (1.00), which indicates that experimental data were close to the predicted data of the model. Additionally, the adjusted R^2^ values of PAH and COD models (0.93 and 0.96, respectively) were in good agreement with the predicted R^2^ values (0.80 and 0.78, respectively). These values of R^2^, adjusted R^2^, and predicted R^2^ verify the adequacy of both models (PAHs and COD) under the experimental conditions. Additionally, in Figure 2, all the values are close to the 45° line, showing a reasonable agreement between experimental and predicted data. The results achieved from the experiments were very close to the predicted results, which proved the significance of the model. This also confirmed the correlation between independent variables and responses.

### 3.3. Effect of Operating Parameters on Responses

**Figure 3:**
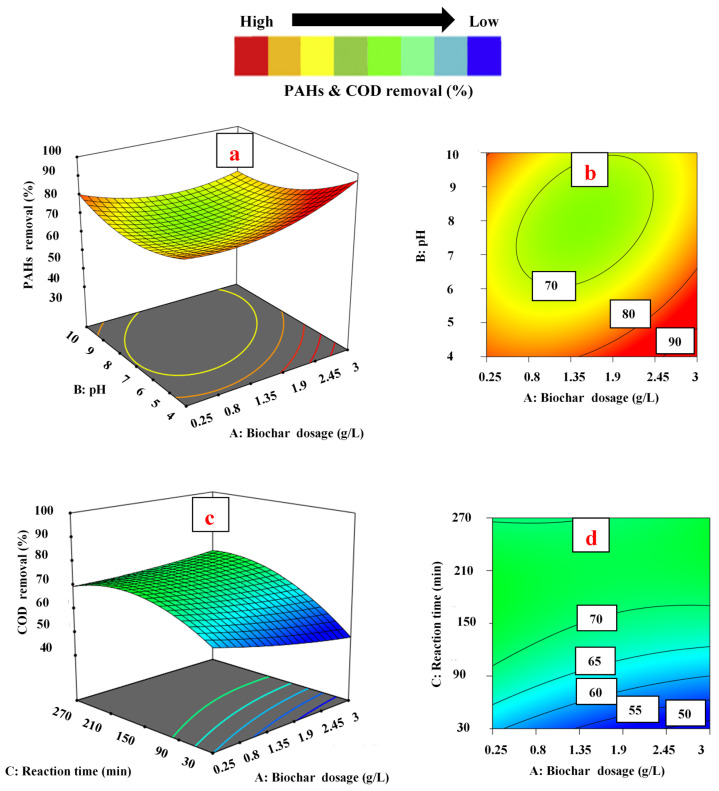
Three-dimensional surface plots for the effect of biochar dosage: (**a**,**b**) and PAH removal (**c**,**d**) and COD removal.

**Figure 4:**
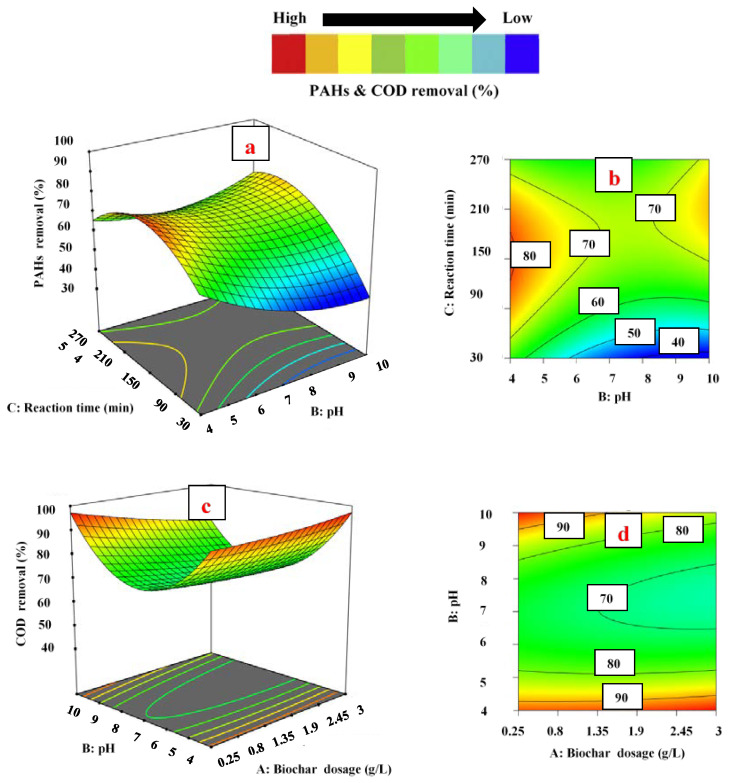
Three-dimensional surface plots for the effect of pH: (**a**,**b**) and PAH removal (**c**,**d**) and COD removal.

**Figure 5:**
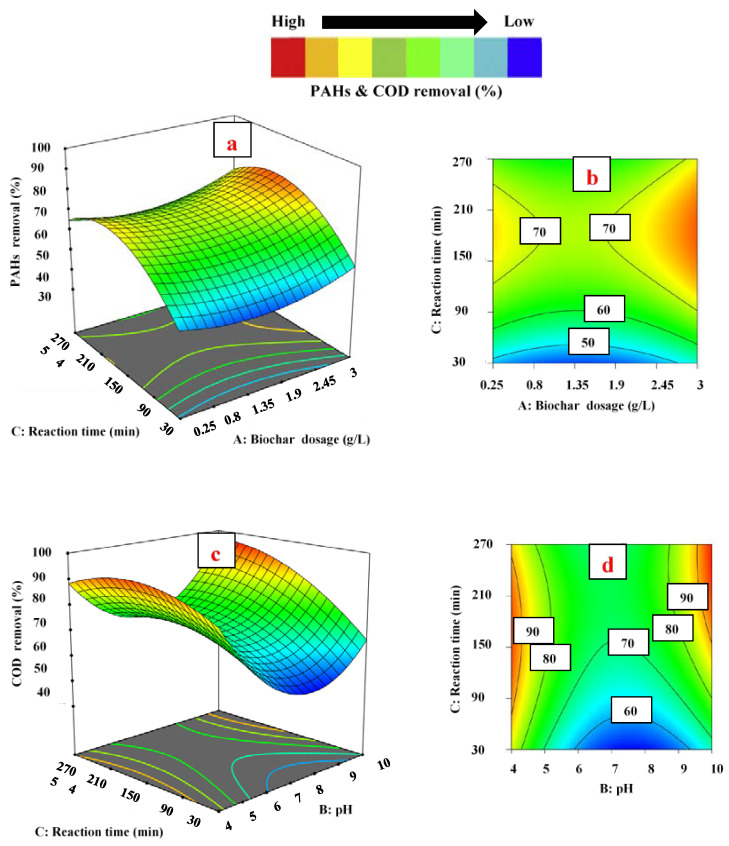
Three-dimensional surface plots for the effect of contact time: (**a**,**b**) and PAH removal (**c**,**d**) and COD removal.

To examine the influence of independent variables on PAH and COD removal, three-dimensional and contour plots developed by Design Expert software were utilized. In this scenario, the values of two parameters varied within the given range, while other independent variables were kept constant, as shown in Figure 3, Figure 4 and Figure 5.

#### 3.3.1. Effect of Biochar Dosage

In Figure 3a,b, the impact of pH and biochar dosage on the removal of PAHs is presented at 150 min of reaction time. The interaction between both parameters was significant, with a *p*-value of 0.0022. The removal of PAHs was increased by increasing the values of both parameters up to a specific range. It was noticed that the removal of PAHs surged from 79.41% to 94.23% when the amount of biochar increased from 0.25 to 3.00 g L^−1^, keeping pH constant at 7.0. It was assumed that the higher concentration of biochar in the aqueous solution enhanced the availability of more active sites on the surface of adsorbents for PAH adsorption. Figure 3c,d shows the effect of reaction time and adsorbent dosage on removal of COD at pH 7.0. A significant correlation was observed between reaction time and adsorbent dosage, with a 0.0070 *p*-value. The removal of COD decreased from 60.48% to 48.10%, with an increase in adsorbent dosage from 0.25 to 3.00 g L^−1^ at a 30 min reaction time. As expected, an increase in the concentration of adsorbent leads to more unsaturated sites available for pollutants. In addition, the increase in biochar dosage caused the agglomeration of absorbent particles, which led to a reduction in diffusion path length and surface area for the adsorption of COD [55]. Interestingly, in this study, two contrary trends were confirmed for PAH and COD removal at the same concentration range of biochar, from 0.25 to 3.00 g L^−1^. By increasing the adsorbent dosage from 0.25 to 3.00 g L^−1^, the PAH removal raised and reached its maximum level, while COD removal decreased and reached the minimum level. PW contains a considerable number of organic pollutants (both +ve and −ve ions), which are measured as COD [56]. It is assumed that all the organic pollutants (which are the part of COD), except PAHs, showed a negative trend due to the agglomeration of absorbent particles.

#### 3.3.2. Effect of pH

The pH of the aqueous solution is a very important parameter in the adsorption process because it controls the adsorption efficiency of the adsorbent by varying the charge on the adsorbent surface [57]. Biochar contains a variety of surface functional groups (mainly oxygen-containing groups, such as carboxylate, -COOH; and hydroxyl, -OH). When the pH of the solution increases, the behavior of these functional groups changes [58]. Most of these functional groups on biochar are protonated at low pH and appear positively charged. For pH < pH_pzc_ (point of zero charge), the biochar surface is positively charged, favoring adsorption of the anions. However, in a basic environment (when pH > pH_pzc_), the surface of the biochar is negatively charged. Thus, biochar can easily capture cations at higher pH levels [59,60].

In Figure 4a,b the impact of pH and contact time on the removal of PAHs is shown at 1.625 g L^−1^ of biochar dosage (fixed). The results exhibited that the removal percentage of PAHs was at its maximum (76.5%) at a low pH (4.0). However, when pH was increased from pH 4.0 to 10.0, the removal of PAHs decreased from 76.5% to 57.7% at 90 min of contact time. The higher removal of PAHs at low pH may be due to the increase in positively charged functional groups on the surface of biochar, which causes higher interaction between the surface of biochar and PAH molecules. Contrarily, an increase in pH resulted in a less positive biochar surface that adsorbed the PAH species at a higher pH. As the pH increases, the positively charged functional groups on the surface of biochar decrease and the negatively charged functional groups increase [60,61]. The positively charged functional groups compete with negatively charged functional groups and interact with the molecules of PAHs on active sites for adsorption, which leads to a reduction in adsorption efficiency of the biochar. A similar trend was reported by Kumar et al. [62], who obtained the maximum removal of phenanthrene (almost 90%) at a low pH (almost 4.0), while removal was decreased (70%) at a higher pH (12.0).

On the other hand, in Figure 4c,d the influence of pH and biochar dosage on COD removal is exemplified at 150 min of contact time (fixed). Initially, at pH 4.0, the COD removal was 71.3% (at 0.25 of adsorbent dosage). The removal percentage declined when the pH value increased from pH 4.0 to 7.0 and reached its minimum level (65.5%). However, as the pH value increased beyond pH 7.0, the COD removal started rising again and reached its maximum level (96.8%) at pH 10.0. The significant removal of COD in both acidic and basic media could be due to the presence of +ve and –ve ions (organic compounds), which adsorbed on negatively/positively charged biochar sites. In comparison, the removal of COD reached its minimum level at pH 7.0 (neutral). A decrease in organics removal could be due to the fact that the net charge at adsorbent sites was zero and the removal of COD at neutral pH occurred by only precipitation [63]. Khursid et al. [6] reported similar results, and obtained minimum removal of COD at neutral pH; while in both acidic and basic media, the remediation was at its maximum. The results of the current study are in contrast with the results of Lam et al. [64], who reported an acidic pH value of 2.0 as the optimum value for maximum removal of COD. They also reported that the removal of COD decreased upon increasing the pH of the solution from pH 2.0 to 11.0. The reason for the contrast in results may be due to differences in the properties of industrial effluents and operating parameter conditions.

#### 3.3.3. Effect of Contact Time

In the adsorption process, contact time/reaction time is an important parameter and plays a vital role in the removal of organic pollutants. Figure 5a,b shows the interaction between reaction time and biochar dosage, while in Figure 5c,d the interaction between reaction time and pH is shown to study the effect of contact time on PAH and COD removal. In Figure 5a,b it can be seen that, at 30 min of reaction time, 48.3% removal of PAHs was attained when the biochar dosage was 0.25 g L^−1^ (fixed). The reduction in PAHs increased as the contact time increased from 30 to 180 min.

The maximum removal of PAH, about 73.8%, was obtained at 180 min of contact time. However, as the reaction time increased beyond 180 min, PAH removal declined and reached 65.2%. A similar trend was also observed for COD removal, as shown in Figure 5c,d; initially, at 30 min of contact time, 79.6% COD reduction was observed. COD removal continuously increased as the reaction time increased from 30 to 180 min and reached a maximum level of about 92.7%. Beyond 180 min of contact time, COD reduction declined. The reason for this trend may be the availability of maximum binding sites at an initial reaction time of 30 min, which enhanced the interaction between adsorbent and organic contaminants. Therefore, the adsorption sharply increased after 30 min of reaction to 140 min of reaction time. After 140 min of shaking time, fewer binding sites were available, and adsorption was slower. At 180 min of contact time, all the sites were saturated, and the remaining pollutants were too arduous to be filled. A decrease in PAH and COD removal after 180 min of reaction time showed the possibility of physical attachment of organic pollutants with the surface or saturation of active sites by chemical adsorption. The associated reasoning for this might be the repellent forces between the adsorbed pollutants and the free pollutants in the solution [65]. In addition, as the contact time increases after equilibrium, some of the adsorbed pollutants might be released into the solution [65]. A similar trend was reported by Sofia and colleagues regarding contact time [66]. They obtained maximum removal of pollutants at 180 min of contact time. A decline was observed in pollutant removal after 180 min of reaction time [66].

### 3.4. Validation of the Model

Validation experiments were performed based on numerically optimized values of operating parameters to confirm the suitability of the model (Equations (3) and (4)). The optimum values of operating parameters were found based on RSM analysis for the highest desirability [38]. In this case, for all independent variables, the “in range option” was targeted, while for the dependent variables (PAH and COD removal), the “maximized option” was chosen. Based on the above conditions, one hundred (100) solutions were generated by the Design Expert software where the predicted optimal conditions of solution number (2) were selected due to the highest desirability value (1.00). In numerical optimization, the predicted optimal conditions of operating parameters were biochar dosage = 2.99 g L^−1^, pH = 4.02, and reaction time = 208. A total of 89 min with the highest desirability (1.00). Under these optimal values of variables, 93.16% of PAHs and 97.84% of COD removal was predicted, as shown in Table 5. Verification experiments were conducted in triplicate with optimal conditions of operating parameters, and the average removal percentage of PAHs and COD was taken. From experiments, 95.34% of PAHs and 98.21% of COD removal was attained, close to predicted values. The small error between predicted and experimental values confirmed the accuracy and the suitability of the model, as shown in Table 5.

It appears from the literature, just few studies have been conducted for the remediation of PAHs from wastewater using different types of biochar. However, mostly studies focused on the removal of PAHs from soil via various kind of biochar. The removal efficiencies of PAHs and COD by other adsorbent are shown in Table 6 in comparison with the current study.

### 3.5. Regeneration Study

**Figure 6:**
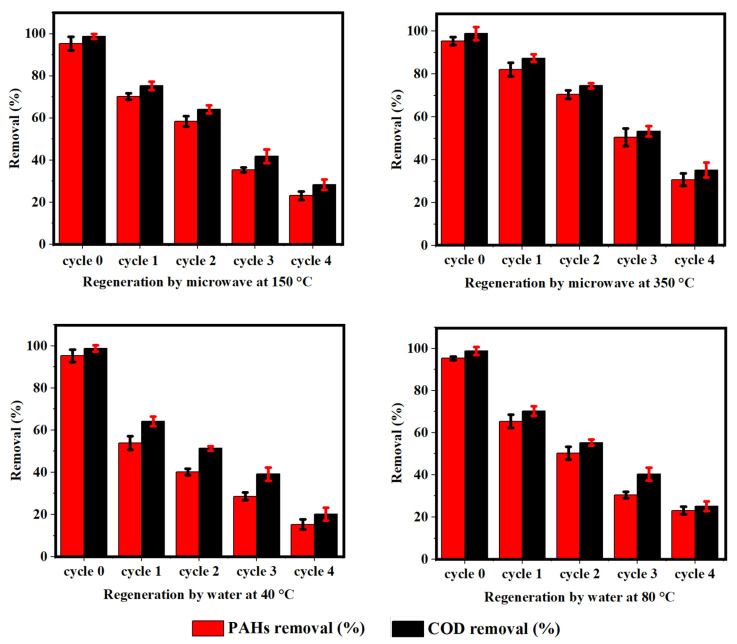
Regeneration study of PKSB for PAH and COD removal.

Regeneration of biochar is essentially a reverse cycle of the adsorption process. Usually, two principals, “adsorbate desorption” and “adsorbate decomposition” are involved in the regeneration process [48]. In the current work, to evaluate the potential of reuse PKSB, a regeneration study was carried out. Two different kinds of treatment, microwave irradiation and water treatment, were assessed on spent PKSB. In Figure 6, the results of four regeneration cycles of spent PKSB are shown. Regeneration by microwave irradiation was better than the water treatment. In microwave regeneration, spent PKSB was regenerated at 150 °C and 350 °C. The complete procedure for microwave regeneration has been discussed elsewhere [49]. Spent PKSB regenerated at 150 °C removed relatively lower PAHs and COD compared to one that was regenerated at 350 °C, as shown in Figure 6. In the first cycle, the removal of PAHs and COD by microwave-regenerated PKSB (at 350 °C) stood at 82.12% and 87.34%, respectively. However, PAH and COD removal values by microwave-regenerated PKSB (at 150 °C) were 70.25% and 75.31%, respectively. Likewise, for water treatment regeneration, the removal of PAHs and COD by water treatment-regenerated PKSB at 40 °C was lower than that observed at 80 °C. PKSB regenerated by water treatment at 40 °C removed 53.85% of PAHs and 64.23% of COD in the first cycle. However, PKSB regenerated by water treatment at 80 °C removed 65.38% of PAH and 70.31% of COD. This indicated that the low temperature (150 °C—microwave and 40 °C—water) was not adequate to recover and repair the PKSB surface properties. It can be seen from Figure 6 that the removal efficiency of PAHs and COD by regenerated PKSB declined in each successive cycle compared to fresh PKSB (cycle 0) for both microwave and water treatment. In the first cycle, the elimination of PAHs and COD by regenerated PKSB (via both microwave and water treatment) was slightly less compared to that of fresh PKSB. Such a reduction in the removal percentages of PAHs and COD was found to continue in each successive period. In the last cycle, the removal of PAHs and COD was recorded in a range of 15.0% to 35.0% for both treatments (microwave and water treatment). The gradual decrease in PAH and COD removal percentages in each consecutive cycle by regenerated PKSB may be due to the destruction of active chemical sites (both +ve and −ve) and the pore structure of PKSB during the water and microwave treatment process [4,50].

## 4. Conclusions

In the present study, the hazardous micro-pollutants PAHs and COD from PW were eliminated by PKSB. This study demonstrated that all three independent variables, biochar dosage, pH, and contact time, significantly influenced the removal of PAHs and COD by PKSB. For optimal experimental conditions of the adsorption process and maximum removal of PAHs and COD, a central composite design based on a response surface methodology was employed. Optimum conditions of the current study were found to be biochar dosage of 2.99 g L^−1^, pH of 4.0, and a contact time of 208.89 min. Under these optimal values of independent variables, 93.16% and 97.84% removal of PAHs and COD were predicted, respectively; however, in the lab, the maximum removal of PAHs and COD under optimal conditions was 95.34% and 98.21%, respectively. The small difference between predicted and experimental values showed a satisfactory agreement between quadratic models and experimental values. In addition, the regenerated PKSB showed significant removal of PAHs and COD from PW. Our results showed that the PKSB under investigation could be a favorable, low-cost, and environmentally friendly adsorbent for both PAH and COD removal from PW. In future, based on this study, it could be used for the treatment of different emerging micro-pollutants from PW and other sources of wastewater.

## Figures and Tables

**Table 1:** Treatment methods used for PAH removal.

Methods	Pollutants	Year	References
Coagulation process	16 PAHs	2021	[11]
Electrocoagulation	PAHs	2021	[12]
Ozonation	16 PAHs	2021	[13]
Heterogeneous Fenton oxidation	15 PAHs	2020	[14]
Electrochemical oxidation	PAHs	2020	[15]
Advanced biological treatment	08 PAHs	2020	[16]
Fenton-like oxidations	16 PAHs	2019	[17]
Nano-filtration membranes	03 PAHs	2019	[18]
Bioremediation	14 PAHs	2019	[19]
UV photo-degradation	08 PAHs	2018	[20]
Ultrasound-Fenton	PAHs	2018	[21]

**Table 2:** Experimental design matrix and the results for removal percentage of PAHs and COD.

No	Factors			PAHs Removal (%)		COD Removal (%)	
	*x*_1_	*x*_2_	*x*_3_				
	Biochar Dosage (g/L)	pH (-)	Contact Time (min)	Actual Responses	Predicted Responses	Actual Responses	Predicted Responses
1	1.62	4.0	150	83.40	80.95	94.60	94.54
2	3.00	10.0	270	77.40	76.55	95.70	95.04
3	1.62	10.0	150	69.30	70.47	91.20	89.40
4	0.25	10.0	270	80.10	81.20	97.80	100.39
5	1.62	7.0	150	70.30	68.38	68.30	69.51
6	3.00	10.0	30	40.20	41.09	55.30	56.93
7	1.62	7.0	150	62.10	68.38	71.30	69.51
8	0.25	4.0	30	62.10	63.27	87.20	88.32
9	0.25	7.0	150	72.10	72.71	73.80	73.02
10	1.62	7.0	270	63.70	61.39	73.10	70.10
11	1.62	7.0	150	70.20	68.38	65.20	69.51
12	1.62	7.0	30	42.30	43.33	52.20	53.34
13	3.00	7.0	150	80.30	78.41	69.20	68.12
14	3.00	4.0	270	79.30	81.93	93.10	95.33
15	0.25	10.0	30	50.00	47.69	80.10	78.33
16	3.00	4.0	30	80.10	79.32	86.00	83.87
17	0.25	4.0	270	64.50	63.93	84.90	83.73

**Table 3:** RSM/ANOVA output table for PAHs and COD.

Source	SS	DF	MS	*F*-Value	*p*-Value	Status
PAHs						
Model	2697.87	9	299.76	25.77	0.0001	Significant
*x*_1_	81.22	1	81.22	6.98	0.0333	
*x*_2_	274.58	1	274.58	23.61	0.0018	
*x*_3_	815.41	1	815.41	70.10	0.0001	
*x*_1_ *x*_2_	256.51	1	256.51	22.05	0.0022	
*x*_2_ *x*_3_	539.56	1	539.56	46.39	0.0003	
*x*_1_^2^	138.11	1	138.11	11.87	0.0108	
*x*_2_^2^	143.94	1	143.94	12.37	0.0098	
*x*_3_^2^	687.64	1	687.64	59.12	0.0001	
Residual	81.42	7	11.63			
Lack of Fit	37.14	5	7.43	0.3354	0.8595	Non-significant
COD						
Model	3113.08	9	345.90	38.06	0.0001	Significant
*x*_1_	60.02	1	60.02	6.60	0.0370	
*x*_2_	66.05	1	66.05	7.27	0.0308	
_X3_	702.24	1	702.24	77.27	0.0001	
*x*_1_ *x*_2_	143.65	1	143.65	15.81	0.0054	
*x*_1_ *x*_3_	128.80	1	128.80	14.17	0.0070	
*x*_2_ *x*_3_	355.11	1	355.11	39.07	0.0004	
*x*_2_^2^	1351.87	1	1351.87	148.75	0.0001	
*x*_3_^2^	162.48	1	162.48	17.88	0.0039	
Residual	63.62	7	9.09			
Lack of Fit	45.01	5	9.00	0.9677	0.5789	Non-significant

**Table 4:** RSM model fit summary output table for PAHs and COD.

Statistical Factors	Abbreviated as	PAHs	COD
Standard deviation	St. Dev.	3.41	3.01
Coefficient of determination	R^2^	0.97	0.96
Mean	Mean	67.49	78.76
Predicted R^2^	Pre. R^2^	0.80	0.78
Adjusted R^2^	Adj. R^2^	0.93	0.95
Coefficient of variance	C.V.%	5.05	3.83
Adequate precision	A.P.	15.61	20.35

**Table 5:** Response results of the experiment and prediction model for optimal mix design.

Dependent Variable	Biochar Dosage. (g L^−1^)	pH	Reaction Time (min)	Predicted Solution	Lab Experiments	Error (%)
PAHs removal (%)	2.99	4.0	208.89	93.16	95.34	2.28
COD removal (%)	2.99	4.0	208.89	97.84	98.21	0.37

**Table 6:** Comparison among the current and previous studies that used biochar for PAH and COD removal.

Adsorbent	Source of Pollutants	Pollutants	Removal (%)	References
Tea waste biochar	Oil and gas wastewater	COD	95.5	[6]
Magnetic wood biochar (Fe_3_O_4_-WB)	Estuary sediment	PAHs	87.0	[65]
Biochar-loading copper ions (Cu-BC)	Constructed wetland	1 PAH	>90	[66]
Rice husk biochar	Municipal wastewater	Organic pollutants/COD	94.0	[67]
Corn straw biochar	Synthetic wastewater	COD	95.4	[68]
Water treatment sludge–derived biochar	Aquatic sediments	3 PAHs	87	[69]
Rice straw-derived biochar	Coking plant soil	PAHs	58.4	[70]
Magnetic biochar from tea waste	Synthetic wastewater	4 PAHs	89	[71]
Municipal waste biochar	Municipal wastewater	COD	90.0	[72]
Palm kernel shell biochar	Produced water	PAHs and COD	95.3 and 98.21	Present study

## Data Availability

Not applicable.
